# Immune checkpoint inhibitor treatment induces colitis with heavy infiltration of CD8 + T cells and an infiltration pattern that resembles ulcerative colitis

**DOI:** 10.1007/s00428-021-03170-x

**Published:** 2021-08-02

**Authors:** Sara Hone Lopez, Gursah Kats-Ugurlu, Remco J. Renken, Henk J. Buikema, Marco R. de Groot, Marijn C. Visschedijk, Gerard Dijkstra, Mathilde Jalving, Jacco J. de Haan

**Affiliations:** 1 Department of Medical Oncology, University Medical Center Groningen, University of Groningen, PO Box 30.001, 9700 RB Groningen, The Netherlands; 2 Department of Pathology and Medical Biology, University Medical Center Groningen, University of Groningen, PO Box 30.001, 9700 RB Groningen, The Netherlands; 3 Department of Biomedical Sciences of Cells & Systems, University Medical Center Groningen, University of Groningen, PO Box 30.001, 9700 RB Groningen, The Netherlands; 4 Cognitive Neuroscience Centre , University Medical Center Groningen, University of Groningen, PO Box 30.001, 9700 RB Groningen, The Netherlands; 5 Department of Hematology, University Medical Center Groningen, University of Groningen, PO Box 30.001, 9700 RB Groningen, The Netherlands; 6 Department of Gastroenterology and Hepatology, University Medical Center Groningen, University of Groningen, PO Box 30.001, 9700 RB Groningen, The Netherlands

**Keywords:** Immune checkpoint inhibitor, CD8-positive T-lymphocytes, Colitis, Inflammatory bowel disease, Acute graft-versus-host disease

## Abstract

Colitis is a common, but poorly understood, adverse event of immune checkpoint inhibitors that are standard-of-care for an expanding range of cancer types. This explorative study aimed to describe the immune infiltrates in the colon from individuals developing checkpoint inhibitor colitis and compare them to well-known immunophenotypes of acute graft-versus-host disease, ulcerative colitis, and Crohn’s disease. Colon biopsies (*n* = 20 per group) of patients with checkpoint inhibitor colitis, acute graft-versus-host disease, ulcerative colitis and Crohn’s disease, all colitis treatment-naïve, and of individuals with a normal colon were analyzed using immunohistochemistry: CD8 for cytotoxic T cells, CD4 for T helper cells, and CD68 to identify cells of macrophage lineage. CD8 + T cell, CD4 + T cell, and CD68 + cell counts were performed. Cell infiltration was scored as scattered/patchy or band-like in the superficial and deep gut mucosa. Checkpoint inhibitor colitis was found to be heavily infiltrated by CD8 + T cells. Comparative analysis between groups showed that both CD8 + T cell counts (*P* < 0.01) and immune cell infiltration patterns in checkpoint inhibitor colitis were most similar to those observed in ulcerative colitis, with a deep band-like CD4 + T cell infiltration pattern and a superficial band-like CD68 + cell infiltration pattern in both. In conclusion, this is the first immunohistopathological study comparing infiltrate characteristics of checkpoint inhibitor colitis, acute graft-versus-host disease, ulcerative colitis, and Crohn’s disease. Checkpoint inhibitor colitis samples are heterogeneous, heavily infiltrated by CD8 + T cells, and show an immune cell infiltration pattern that is more similar to ulcerative colitis than to colonic acute graft-versus-host disease or colonic Crohn’s disease.

**Supplementary Information:**

The online version contains supplementary material available at 10.1007/s00428-021-03170-x.

## Introduction

Immune checkpoint inhibitor (ICI) therapy has revolutionized cancer treatment [1]. ICIs enhance the anti-cancer immune response by inhibiting the cytotoxic T-lymphocyte-associated protein 4 (CTLA-4) or by inhibiting the programmed cell death protein 1/programmed death-ligand 1 (PD-1/PD-L1) pathways that have a physiological role in preventing autoimmunity [1, 2]. Colitis is the most fatal immune-related adverse event of both anti-CTLA-4 and combined anti-CTLA-4 and anti-PD-1/PD-L1 therapy [3, 4]. The risk of developing grade III–IV checkpoint inhibitor colitis (CIC) in patients receiving CTLA-4 inhibitors, PD-1/PD-L1 inhibitors, or the combination is 8%, 1–2%, and 11%, respectively [5–7]. Management of CIC is grade-dependent and based on therapeutic strategies used in inflammatory bowel disease (IBD). First-line treatment consists of mesalazine or corticosteroids; second-line treatment involves immunosuppressants like infliximab [8, 9]. However, not all patients respond to this treatment strategy [3, 9]. Furthermore, second-line immunosuppressants may dampen the anti-tumour response and have been associated with worse overall survival in patients with severe immune-related adverse events [9, 10]. The pathophysiology of CIC is not completely understood. The histology of CIC resembles the well-known phenotypes of acute graft-versus-host disease (aGVHD) colitis and IBD [11–14]. Cytotoxic, helper, and regulatory T cells and macrophages are involved in the anti-tumour effects of ICIs and are also important in the development of aGVHD and IBD [15–19]. The role of these cells in CIC is unclear.

A detailed comparison of CIC with better understood colitis subtypes may provide insight into its immunopathology. In the present study, we compared immunohistochemical phenotypes of immune infiltrates in colonic mucosa biopsies from colitis treatment-naïve individuals with CIC, aGVHD colitis, ulcerative colitis (UC), Crohn’s disease (CD), and control samples.

## Materials and methods

### Patient selection

Archival formalin-fixed paraffin-embedded colitis treatment-naïve colon biopsies from routine diagnostic procedures from patients diagnosed with histologically confirmed CIC, aGVHD, UC, or CD above 18 years of age were retrieved. Biopsies from all 20 individuals diagnosed with CIC between 2010 and 2018 at the University Medical Center Groningen (UMCG) that met the inclusion criteria were enrolled. For all other groups, 20 consecutive patients were included. Diagnosis of IBD was based on endoscopic and histopathological findings, in accordance with the European Crohn’s and Colitis Organisation (ECCO) and the European Society of Gastrointestinal and Abdominal Radiology (ESGAR) guidelines [20, 21]. The control group consisted of 20 individuals who had a diagnostic colonoscopy but in whom no endoscopic or histopathological abnormalities were detected.

The biopsies were obtained and used for diagnostic purposes between 1996 and 2018. This led to differences in the number of biopsies taken per patient, as well as in quality and size. One biopsy per patient was selected in order to investigate the same amount of tissue per patient. To ensure that the biopsies included in the study were representative and comparable, the following criteria were applied.

First, biopsies were selected and reviewed if presence of inflammation had been established during routine diagnostic procedures. Next, the selected biopsies were assessed for tissue completeness (i.e. presence or absence of mucosal layers) and quality (e.g. tissue integrity and size). Those biopsies showing mucosal denudation and/or ulceration were excluded. Next, we assessed the selected biopsies for completeness (i.e. presence or absence of surface epithelium, lamina propria, and muscularis mucosae). We excluded those samples showing mucosal denudation and/or ulceration. When possible, choice of biopsy was also based on location to obtain an even number of right- and left-sided biopsies. The requirement for informed consent was exempted given that the biopsies were archival and obtained during routine diagnostic procedures. Individuals were excluded from the study in case of registered objection for use of their tissue for research purposes. This study was approved by the UMCG Medical Ethics Committee and registered in The Netherlands Trial Register (NL8135).

### Immunohistochemical staining

CD8, CD4, and CD68 immunohistochemistry was performed according to standard protocols on 4-μm FFPE tissue sections (Supplementary Table 1). Tonsil tissue was used as positive and negative (no primary antibody) control for all antibodies. Haematoxylin and eosin (HE) staining was performed using Tissue-Tek Prisma Plus and Film Automated Slide Stainer and Coverslipper (Sakura Finetek Europe V, Alphen a/d Rijn, The Netherlands) according to the manufacturer’s instruction.

### Immunohistochemical analysis

**Fig. 1:**
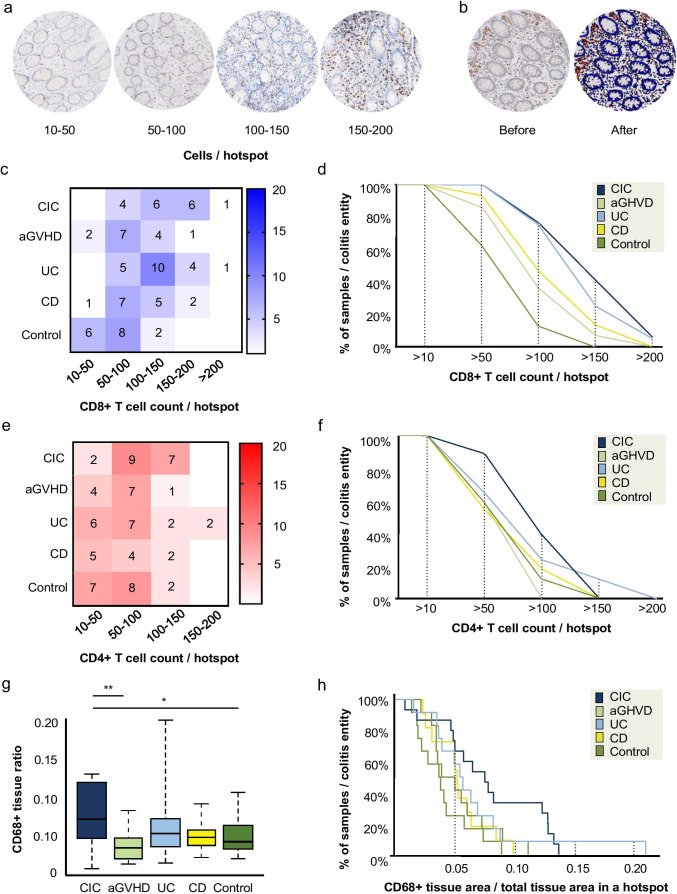
Distribution of CD8 + T cell and CD4 + T cell counts and CD68 + tissue ratios between samples in colitis groups and controls. **a** Representative examples of hotspots (0.24 mm^2^) of CD8 + T cell staining with counts of 10–50 cells, 50–100 cells, 100–150, and 150–200 cells at a × 400 magnification. **b** Representative example of hotspot illustrating quantification of CD68 + tissue ratio before and after tissue processing with Visiopharm Software. Software recognition of CD68-positive cell surface (red) and CD68-negative cell surface (blue) was based on the shape and size of the cells present, in combination with the intensity of the staining. The threshold of CD68 staining intensity for positive tissue was determined manually, as recognition of the CD68 + staining used has not yet been automatized. The same staining intensity threshold was used for all biopsies. CD68 + staining was quantified using the CD68 + tissue ratio per hotspot. This was determined by dividing CD68 + cell surface by total cell surface of the hotspot. In the example shown above, the CD68 + tissue ratio was 0.035 (0.0085 mm^2^ CD68 + tissue/0.2404 mm^2^ total hotspot cell surface. **c** Heatmap illustrating CD8 + T cell count frequencies per group. The intensity of the colour reflects the number of samples within each group with a specific cell count range. **d** CD8 + T cell counts per hotspot between colitis groups and the control group, displayed as cumulative proportion of samples per colitis entity having a cell count higher or equal for each cell count (> 10, > 50, > 100, > 150, and > 200 cells/per hotspot). **e** Heatmap showing CD4 + T cell count frequencies per sample per group. **f** Cumulative proportion of samples per colitis entity or control group having a CD4 + T cell count higher or equal for each cell count. **g** Boxplot illustrating CD68 + tissue ratio frequencies per group**.** Significant differences between groups are indicated (*P < 0.05; **P < 0.01). **h** Cumulative proportion of samples per colitis entity or control group having a CD68 + tissue ratio higher or equal for each CD68 + tissue ratio

Samples were digitalized using Philips Ultra-Fast Scanner (Philips Digital Pathology Scanner, Best, The Netherlands) and viewed using Philips Image Management System Pathology Case Viewer software. Initial evaluation of the immune infiltrate revealed a tendency towards a “patchy” distribution—i.e. focal areas with clearly higher density of the infiltrate. To ensure a representative selection of the highest immune cell count per sample, the following methodology was applied. First, the biopsy was evaluated as a whole at 100 × magnification. Based on this initial evaluation, three equally sized areas with the highest density of the cell of interest were selected—these areas of 0.24 mm^2^ are from here onwards referred to as hotspots. Next, a visual comparison of the three hotspots was performed and the hotspot with the highest density of cells of interest was identified. The immune infiltrate in the selected hotspot was then quantified manually. Areas with > 20 clustered lymphoid cells or lymphoid follicles were excluded; CD8 + T cell and CD4 + T cell counts were expressed in ranges of 0–10, 10–50, 50–100, 100–150, 150–200, and > 200 cells per hotspot (Fig. 1a). CD4 + staining showed background staining in most slides. This was corrected through image enhancement (IE). CD68 + staining showed CD68 + cell clustering, background and pseudopodia staining in most samples. Even after IE, individual cell identification was impossible. Therefore, CD68 + stainings were quantified using the ratio of CD68 + tissue surface to total tissue surface per hotspot. CD68 + tissue ratio was determined using Visiopharm Software (version 2020.02.0.7219, Hørsholm, Denmark) (Fig. 1b). Infiltration patterns of CD8 + T cell, CD4 + T cell, and CD68 + cells in the mucosa were also investigated (× 400 magnification). While evaluating infiltration patterns, two predominant cell infiltration patterns were noticed: scattered/patchy (unorganized distribution of solitary cells sometimes accompanied by the presence of small clusters) and band-like (organized distribution of solitary cells and/or of small clusters showing infiltrate continuity). A distinction was made between infiltration patterns in the superficial and deep mucosal layers. The most prominent cell infiltration pattern in the superficial and deep mucosa per biopsy was described. Cell count and infiltration patterns limited to lamina propria excluding surface and crypt epithelium and submucosa were scored manually in a randomized fashion by two blinded observers (S. H.L., G. K-U). HE slides were used to assess the following morphological features: presence of non-necrotizing granuloma in Crohn’s disease cases; presence of apoptotic bodies, intraepithelial lymphocytes, cryptitis, crypt loss or damage, crypt abscesses, crypt distortion, and irregular basal membrane in the CIC group. In addition, HE slides served as a reference for the tissue structure and composition during both cell count and infiltrate pattern analysis. Slides in which the muscularis mucosae was absent, or insufficient tissue quality or quantity were excluded. In case of result discrepancy (< 5% of all analyses), samples were re-analyzed and discussed until consensus was reached.

### Statistical analysis

Statistical analysis was performed using SPSS (version 25, IBM, New York, NY), MATLAB (version 17.0, MathWorks, Natick, MA) and R Studio (version 1.2.5033, PBC, Boston, MA). Age is presented as median and corresponding range per group while categorical variables are presented in percentages. Differences in age and sex between the groups were determined using one-way ANOVA with post hoc Tukey test. The Spearman rank-order correlation test with an uncorrected *p* value was used to study correlations. To represent the distribution of CD8 + T cell and CD4 + T cell counts and CD68 + tissue ratio, the cumulative distribution (Fc) was determined, scaled (sFc, maximal value equals 1), and inverted, i.e. *F* = 1-sFc, per hotspot per group. In order to identify differences or similarities between groups, the corresponding distributions were compared. First, for each cell count range as well as for the CD68 + tissue ratio, the difference between two inverted cumulative distributions was calculated. Next, this difference was summed across all cell count ranges or CD68 + tissue ratios, forming the group difference (Δ). To analyze statistical differences between CIC and each of the other four groups for cell counts or CD68 + tissue ratios, permutation testing was applied under the null hypotheses that the distribution is equal. For each comparison (CIC vs UC, CIC vs CD, etc.) the labels were randomly permuted 1000 times. To identify the group CIC is most similar to, its similarity with the other four groups was calculated for both CD8 + T cell and CD4 + T cell counts. Cell count similarity was measured using the following formula: *S* = 1-abs(Δ)/4. For CD68 + tissue ratio similarity, the formula was *S* = 1-abs(Δ). Next, permutation testing (*n* permutations per comparison = 1000) was applied under the null hypothesis that all groups were equal (i.e. their labels can be freely permuted under the null hypothesis). For the analysis of CD8 + T cell, CD4 + T cell, and CD68 + T cell infiltration patterns, a binary code was implemented. ‘Zero’ representing a scattered/patchy infiltration pattern or ‘one’ representing a band-like infiltration pattern was attributed to each staining in both the superficial and deep mucosal layers of each sample (Supplementary Table 2). This allowed for divisive hierarchical clustering analysis using Jaccard’s distance, thus facilitating descriptive analysis of intra- and inter-group variation.

## Results

### Patient characteristics

Patient characteristics at time of colonoscopy are presented in Table 1. In summary, most individuals in the aGVHD, CD, and control group were women. Patients with CIC and aGVHD were older than patients with UC, CD, and controls. In the CIC group, most patients were treated for melanoma. In the aGVHD group, most had been treated for acute myeloid leukaemia.

**Table 1:** Patient characteristics

	**CIC *n* (%)**	**aGVHD *n* (%)**	**UC *n* (%)**	**CD *n* (%)**	**Control *n* (%)**
**Gender**					
Male	11 (55%)	8 (40%)	11 (55%)	4 (20%)	3 (15%)
Female	9 (45%)	12 (60%)	9 (45%)	16 (80%)	17 (85%)
**Age**^**1,2**^	56 (39–78)	55 (29–66)	38 (18–73)	31 (18–56)	47 (18–68)
**Biopsy location**					
Left colon	10 (50%)	16 (80%)	15 (75%)	8 (40%)	2 (10%)
Right colon	8 (40%)	3 (15%)	2 (10%)	8 (40%)	6 (30%)
Unspecified	2 (10%)	1 (5%)	3 (15%)	4 (20%)	12 (60%)
**Underlying disease**					
Melanoma	16 (80%)				
NSCLC	3 (15%)				
RCC	1 (5%)				
AML		11 (55%)			
ALL		3 (15%)			
Lymphoma		3 (15%)			
Multiple myeloma		2 (10%)			
MDS		1 (5%)			
**Type of ICI**					
Anti-CTLA-4	12 (60%)				
Anti-PD-1	8 (40%)				
**CTCAE CIC clinical grade**					
Grade II	2 (10%)				
Grade III	10 (50%)				
Grade IV	2 (10%)				
Unspecified	6 (30%)				
**aGVHD histological grade**					
Grade I		4 (20%)			
Grade II		16 (80%)			
**Non-necrotizing granuloma**					
Presence				9 (45%)	
Absence				10 (50%)	
Not evaluable				1 (5%)	

^1^Patients with CIC and aGVHD were older compared to patients with UC (*P* < 0.01 and *P* < 0.05, respectively) and CD (*P* < 0.01 and *P* < 0.01, respectively). CIC individuals were also older than controls (*P* < 0.05). ^2^Median in years (range). *CIC* immune checkpoint inhibitor–induced colitis, *aGVHD* acute graft-versus-host disease, *UC* ulcerative colitis, *CD* Crohn’s disease, *NSCLC* non-small cell lung cancer, *RCC* renal cell carcinoma, *AML* acute myeloid leukaemia, *ALL* acute lymphocytic leukaemia, *MDS* myelodysplastic syndrome, *ICI* immune checkpoint inhibitor, *CTLA-4* cytotoxic T-lymphocyte-associated protein 4, *PD-1* programmed cell death protein 1, *CTCAE* Common Terminology Criteria for Adverse Events

All biopsies from patients with CIC showed apoptotic bodies and intraepithelial lymphocytes. Other prevalent morphological features in CIC patients were cryptitis (18/20 patients), crypt loss or damage (17/20 patients), and/or crypt abscesses (15/20 patients). Less common features were crypt distortion (11/20 patients) and an irregular basal membrane (6/20 patients; Table 2).

**Table 2:** Morphological features seen in CIC samples

	**Apoptotic bodies**	**Intraepithelial lymphocytes**	**Cryptitis**	**Crypt loss/damage**	**Crypt abcess**	**Crypt distorsion**	**Irregular basal membrane**
1	Yes	Yes	Yes	Yes	Yes	Yes	No
2	Yes	Yes	Yes	Yes	Yes	Yes	No
3	Yes	Yes	Yes	Yes	Yes	Yes	No
4	Yes	Yes	Yes	Yes	Yes	Yes	No
5	Yes	Yes	Yes	Yes	Yes	Yes	No
6	Yes	Yes	Yes	Yes	Yes	Yes	No
7	Yes	Yes	Yes	Yes	Yes	Yes	No
8	Yes	Yes	Yes	Yes	Yes	Yes	No
9	Yes	Yes	Yes	Yes	Yes	No	Yes
10	Yes	Yes	Yes	Yes	Yes	No	Yes
11	Yes	Yes	Yes	Yes	Yes	No	Yes
12	Yes	Yes	Yes	Yes	Yes	No	No
13	Yes	Yes	Yes	Yes	Yes	No	No
14	Yes	Yes	Yes	Yes	Yes	No	No
15	Yes	Yes	Yes	Yes	Yes	No	No
16	Yes	Yes	Yes	Yes	No	No	Yes
17	Yes	Yes	Yes	No	No	Yes	Yes
18	Yes	Yes	Yes	No	No	No	No
19	Yes	Yes	No	Yes	No	No	No
20	Yes	Yes	No	No	No	No	Yes
Total *n* (%)	**20 (100%)**	**20 (100%)**	**18 (90%)**	**17 (85%)**	**15 (75%)**	**11 (55%)**	**6 (30%)**

### CD8 + T cell counts in CIC are most similar to UC

A significant number of biopsies not being fully evaluable due to the quality, size, and integrity of the available tissue were excluded. Biopsy size was considered unsatisfactory if there was insufficient tissue for 3 hotspots of 0.24 mm^2^ to be selected. This resulted in eighty-two samples with CD8 + and 75 with CD4 + staining being available for cell count analysis, and 62 samples for CD68 + tissue ratio analysis. CD8 + T cell count in CIC was higher than the control group (*P* < 0.05).

When analyzing similarity between all groups, CD8 + T cell counts of CIC were most similar to that of UC (*P* < 0.01; Fig. 1c and d). For CD4 + T cell counts, no differences or similarities between CIC and the other groups were observed (Fig. 1e and f). The CD68 + tissue ratio in CIC increased compared to aGVHD (*P* < 0.01) and controls *P* < 0.05 (Fig. 1g and h).

### CIC shares similarities in CD8 + T cell, CD4 + T cell, and CD68 + cell infiltration patterns with UC

**Fig. 2:**
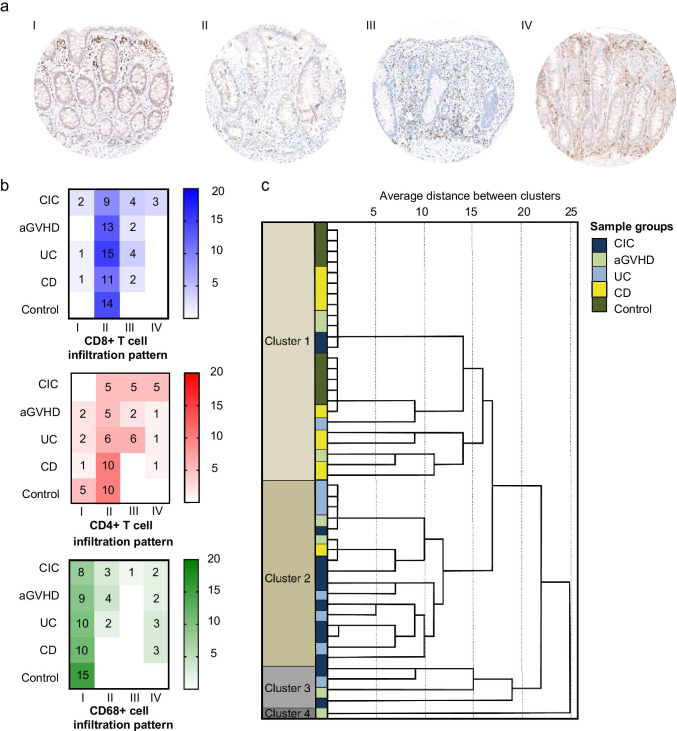
Infiltration patterns of CD8 + T cell, CD4 + T cell, and CD68 + cells. **a** Representative examples of the four infiltration patterns I–IV at × 400 magnification: (I) superficial band-like, deep scattered/patchy; (II) superficial and deep scattered/patchy; (III) superficial scattered/patchy and deep band-like; (IV) superficial and deep band-like. **b** Heatmaps illustrating CD8 + T cell, CD4 + T cell, and CD68 + cell infiltration pattern frequencies per group. The intensity of the colour reflects the number of samples within each group having a specific infiltration pattern. **c** Dendrogram illustrating similarities between individual CIC, aGVHD, UC, CD, and control samples based on CD8 + T cell, CD4 + T cell, and CD68 + cell infiltration patterns. Distance between samples is expressed in the *x*-axis with a smaller distance indicating more similarity between samples. Four different clusters are identified. All samples are colour-coded according to the colitis or control group they belong to: CIC in dark blue, aGVHD in light green, UC in light blue, CD in yellow, and controls in dark green

Several samples were excluded due to absence of muscularis mucosae, or insufficient tissue integrity or quality. For infiltration pattern analyses, 81 samples with CD8 + T cell, 67 with CD4 + T cell, and 72 with CD68 + cell staining were available. Combining the location of the infiltrate in the mucosa (superficial or deep layer) and the pattern (scattered/patchy or band-like), we observed four distinct infiltration patterns: (I) superficial band-like and deep scattered/patchy; (II) superficial and deep scattered/patchy; (III) superficial scattered/patchy and deep band-like; (IV) superficial and deep band-like (Fig. 2a).

In all control samples, all CD8 + T cells and most CD4 + T cells (67%) showed pattern II. All CD68 + cells and some of CD4 + T cells (33%) showed pattern I. These findings were in accordance with previous reports [22]. Colitis groups, particularly CIC, showed more variation of infiltration patterns both in deep and superficial mucosa (Fig. 2b). We assessed inter-individual (variation between patients) and inter-group variation in mucosal infiltration patterns of CD8 + T cell, CD4 + T cell, and CD68 + cells. Only samples with available staining of both deep and superficial mucosa were included in the analysis (*n* = 46, Supplementary Table 2). In the resulting dendrogram, three major clusters were observed (Fig. 2c). Cluster I was formed primarily by controls and CD patients. Cluster II was formed by CIC and UC patients. Cluster III was small and heterogeneous and included two CIC, one aGVHD and one UC sample. Samples in cluster I were characterized by a superficial and deep scattered/patchy CD8 + T cell infiltration pattern, a deep scattered/patchy CD4 + T cell infiltration pattern and a superficial band-like CD68 + cell infiltration pattern. In cluster II, samples shared a deep band-like CD4 + T cell infiltration pattern and a superficial band-like CD68 + cell infiltration pattern. Cluster III samples shared a superficial scattered/patchy CD8 + T cell, CD4 + T cell, and CD68 + cell infiltration pattern. The main difference between CIC samples in clusters II and III was the band-like vs. scattered/patchy superficial infiltration pattern of CD68 + cells (Table 3).

**Table 3:** Predominant patient characteristics and infiltration pattern per cluster

	**Predominant patient group**	**Predominant infiltration pattern**
Cluster I	CD and controls	- CD8 + T cell: superficial and deep scattered/patchy - CD4 + T cell: deep scattered/patchy - CD68 + cell: superficial band-like
Cluster II	CIC and UC	- CD4 + T cell: deep band-like - CD68 + cell: superficial band-like
Cluster III	CIC and aGVHD	- CD4 + T, CD8 + T, and CD68 + cell: superficial scattered/patchy

*CIC* immune checkpoint inhibitor–induced colitis, *aGVHD* acute graft-versus-host disease, *UC* ulcerative colitis, *CD* Crohn’s disease

No differences in CD8 + T cell or CD4 + T cell counts; CD68 + tissue ratio; and CD8 + T cell, CD4 + T cell, and CD68 + cell infiltration patterns were observed between the five anti-CTLA-4 nor the seven anti-PD-1 colitis samples, nor between responders and non-responders to either ICIs or corticosteroids (Supplementary Table 2). No clear differences in cell counts nor infiltration patterns were found between left- and right-sided biopsies (data not shown). Weak positive correlations were observed seen between CD8 + T cell counts and CD4 + T cell infiltration pattern (*r*_s_ = 0.377, *p* < 0.01), CD8 + T cell counts and infiltration pattern (*r*_s_ = 0.309, *p* < 0.01), and CD8 + T cell infiltration patterns and CD68 + cell infiltration pattern (*r*_s_ = 0.368, *p* < 0.05), All other correlations were not significant or had a *r*_s_ below 0.3.

## Discussion

This exploratory study shows heavy infiltration of CD8 + T cells and heterogeneity in CD8 + T cell, CD4 + T cell, and CD68 + cell distribution patterns in colon biopsies of patients with CIC. Infiltrate characteristics in patients with CIC were more similar to UC than to aGVHD or CD.

CD8 + T cells are the effectors of ICI anti-tumour effects, and high levels of CD8 + T cell infiltration in tumours before and during ICI treatment are associated with improved ICI efficacy [23]. The extent of CD8 + T cell mucosal infiltration in CIC reported in this study is comparable to heavy CD8 + T cell infiltration previously described in non-intestinal sites of autoimmune inflammation [24–27].

CIC has been reported to be heterogeneous in terms of immunohistological features (e.g. neutrophil- vs. a lymphocyte-predominant infiltrate) [8, 11–14]. We demonstrate that despite the different aetiologies, UC and CIC share similar CD8 + T cell infiltrates with regard to both cell counts and distribution patterns. Interestingly, both ICI targets PD-1/PD-L1 and CTLA-4 play a role in the pathophysiology of IBD, which may partially account for similarities in the immune phenotype observed between these two colitis forms [28, 29]. No significant similarities or differences between CD4 + T cells and CD68 + macrophage infiltrates in CIC and aGVHD or IBD were observed. The use of one standardized disease severity grading system (such as the Geboes Score) for all colitis groups was dismissed as validity of scoring systems are disease-specific and would have led to inaccurate results.

Three other studies have directly compared CIC to IBD. In line with our findings, Coutzac et al. described no significant differences in regulatory CD4 + T cell infiltrates between IBD and CIC; however, CD8 + T cell infiltrates were not compared [30]. In contrast to our findings, Lo et al. described that IBD samples showed higher CD8 + T cell and CD4 + T cell counts and similar CD68 + cell counts when compared to PD-1 CIC samples, as well as similar CD8 + T cell and CD4 + T cell counts and lower CD68 + cell counts when compared with CTLA-4 CIC [31]. The limited number of IBD samples without distinction between UC and CD in the two abovementioned studies may account for the difference in findings. Adler et al. compared CTLA-4 CIC to UC and observed no differences regarding CD8 + and CD4 + cell infiltrate density between groups [32].

In the present study, immune infiltrates of both CTLA-4 and PD-1 CIC samples were found to be heterogeneous. We observed no clear differences between CTLA-4- and PD-1-inhibitor-induced colitis samples. In contrast, Coutzac et al. described CD8 + T cells to be predominant in the immune infiltrate of PD-1-induced colitis and CD4 + T cells in CTLA-4 colitis. Conversely, Lo et al. reported CTLA-4-induced colitis to have significantly higher CD8-, PD-1-, PD-L1-, and CD68-positive cell counts than PD1/PD-L1-induced colitis. This raises the question whether CIC can be further subtyped, and whether such differentiation is relevant for treatment. As illustrated by our hierarchical cluster analysis, while most CIC samples are closest to UC, others resemble CD or aGVHD. Potentially, patients with CIC showing UC-like infiltrate characteristics may benefit from therapies used in UC, while those with aGVHD-like infiltrate characteristics may benefit from agents used in aGHVD.

In order to better understand and characterize CIC, future studies should include a larger number of patients and expand histopathological analyses to other immune cell populations including intraepithelial lymphocytes or molecular targets such as PD-1. Comparison of CIC to other intestinal diseases beyond IBD and aGVHD, such as microscopic colitis or common variable immunodeficiency (CVID), should be considered to obtain more insight in the CIC phenotype, with specific attention to selecting a uniform histological scoring system. In addition, the effects of biopsy location and timing of the biopsy in relation to start of ICI therapy on inflammatory infiltrate should be explored.

Corticosteroids play a central role in the management of CIC. Although most patients with severe CIC initially respond to high-dose corticosteroids, around one-third either fail to respond or experience relapse [3]. Currently, patients resistant to corticosteroids are often treated with infliximab [3, 9]. In addition to being effective in treating colitis, evidence in mice points towards TNF blockade enhancing the anti-tumour effects of ICI by increasing infiltration of tumour-specific T cells in the tumour and decreasing activation-induced cell death in CD8 + T cells [33]. Another option for corticosteroid-resistant individuals is vedolizumab, a gut-specific immunomodulatory agent applied in IBD that targets integrin α4β7 [34]. A retrospective study suggested that patients treated with infliximab or vedolizumab directly after diagnosis of CIC have better clinical outcomes than those first treated with corticosteroids [9, 35].

The observation that CIC is often heavily infiltrated by CD8 + T cell and CD4 + T cells raises the question whether T cell–selective agents including tacrolimus, cyclosporine, or ustekinumab that are used in aGVHD and IBD may be beneficial in CIC treatment [36–38]. Favourable effects of these agents have been reported in steroid-refractory patients with CIC [8, 39–41]. Given that the indications for ICI therapy are expanding, a larger number of patients with steroid-refractory CIC can be anticipated. This highlights the need to optimize current treatment strategies.

In conclusion, this study is the first to directly compare the infiltrate characteristics of CIC, aGVHD, UC, and CD immunohistochemically. CIC shows heterogeneous phenotypes, heavy infiltration of CD8 + T cells, and strongest resemblance to UC.

## Supplementary Information

Below is the link to the electronic supplementary material.

Supplementary file1 (DOCX 32 KB)

## Data Availability

The datasets generated and analyzed during the current study are not publicly available as they contain information that could compromise research participant privacy/consent. The data can be provided by the corresponding author upon reasonable request**.**
