# Supplementary material for: Immune checkpoint inhibitor treatment induces colitis with heavy infiltration of CD8 + T cells and an infiltration pattern that resembles ulcerative colitis
Source: Virchows Arch. 2021 Aug 2;479(6):1119–29. doi: 10.1007/s00428-021-03170-x (PMC8724151; doi:10.1007/s00428-021-03170-x)
Supplement: Supplementary file 1 — Supplementary file1 (DOCX 32 KB) [file 428_2021_3170_MOESM1_ESM.docx]

**Supplementary Information**

**Immune checkpoint inhibitor treatment induces colitis with heavy infiltration of CD8+ T cells and an infiltration pattern that resembles ulcerative colitis**

**Authors:** Sara Hone Lopez^1^, Gursah Kats-Ugurlu^2^, Remco J. Renken^3,4^, Henk J. Buikema^2^, Marco R. de Groot^5^, Marijn C. Visschedijk^6^, Gerard Dijkstra^6^, Mathilde Jalving^1^, Jacco J. de Haan^1^.

University of Groningen, University Medical Center Groningen, Departments of ^1^Medical Oncology, ^2^Pathology and Medical Biology, ^3^Biomedical Sciences of Cells & Systems, ^4^Cognitive Neuroscience Centre, ^5^Hematology and ^6^Gastroenterology and Hepatology, PO Box 30.001, 9700 RB, Groningen, The Netherlands.

**Corresponding author:** Jacco de Haan, University of Groningen, University Medical Centre Groningen, Department of Medical Oncology, PO Box 30.001, 9700 RB Groningen, The Netherlands, [j.j.de.haan@umcg.nl](mailto:j.j.de.haan@umcg.nl), Tel.+31503612821, Fax +31503614862.

**List of supplementary information available, listed in order of appearance in the main text:**

**Supplementary Table 1.** Specifications of the immunohistochemical stainings used.

**Supplementary Table 2.** Individual infiltration patterns observed per mucosal layer per cell type as included in the hierarchical clustering analysis.

.

**Supplementary Table 1**

|  | **Antibody specifications** | **Antigen retrieval** | **Antibody concentration** | **Antibody incubation** | **Secondary antibodies used** |
| --- | --- | --- | --- | --- | --- |
| **Primary antibodies** | anti-CD4 clone 4B12 (Code M7310) | 80 ͦ C overnight in 0.1 M Tris/HCl buffer pH 9.0 | 1:25 | Overnight 4 ͦ C | RAMpo GARpo |
|  | anti-CD8 clone C8 (Code M7103) |  | 1:25 | 2h at room temperature | RAMpo, GARpo RAGpo |
|  | anti-CD68 clone KP1 (Code M0814) |  | 1:50 | 1h at room temperature | RAMpo GARpo |
| **Secondary antibodies** | Polyclonal rabbit  anti-mouse immunoglobulins/PO, RAMpo (Code P0161) | - | 1:100 | 30 min at room temperature | - |
|  | Polyclonal goat  anti-rabbit immunoglobulins/PO, GARpo (Code P0448) | - |  |  | - |
|  | Polyclonal rabbit  anti-goat immunoglobulins/PO, RAGpo (Code P0160) | - |  |  | - |

Antibody specifications. Primary antibodies were diluted in phosphate-buffered saline (PBS) (0.01 M phosphate buffer solution; 0.15 M NaCl, pH 7.4) with 1% bovine serum albumin (BSA). Secondary antibodies were diluted in PBS-1% BSA with 1% human AB plasma (AB serum). All antibodies used were from DAKO (Glostrup, Denmark).

**Supplementary Table 2**

| **Cluster** | **Group** | **CD8+ T cells** | | | | **CD4+ T cells** | | | | **CD68+ cells** | | | |
| --- | --- | --- | --- | --- | --- | --- | --- | --- | --- | --- | --- | --- | --- |
|  |  | **Superficial** | | **Deep** | | **Superficial** | | **Deep** | | **Superficial** | | **Deep** | |
| 1 | Control | 0 | | 0 | | 0 | | 0 | | 1 | | 0 | |
| 1 | Control | 0 | | 0 | | 0 | | 0 | | 1 | | 0 | |
| 1 | Control | 0 | | 0 | | 0 | | 0 | | 1 | | 0 | |
| 1 | Control | 0 | | 0 | | 0 | | 0 | | 1 | | 0 | |
| 1 | CD | 0 | | 0 | | 0 | | 0 | | 1 | | 0 | |
| 1 | CD | 0 | | 0 | | 0 | | 0 | | 1 | | 0 | |
| 1 | CD | 0 | | 0 | | 0 | | 0 | | 1 | | 0 | |
| 1 | CD | 0 | | 0 | | 0 | | 0 | | 1 | | 0 | |
| 1 | aGVHD | 0 | | 0 | | 0 | | 0 | | 1 | | 0 | |
| 1 | aGVHD | 0 | | 0 | | 0 | | 0 | | 1 | | 0 | |
| 1 | ICI/PD-1 | 0 | | 0 | | 0 | | 0 | | 1 | | 0 | |
| 1 | ICI/PD-1^C^ | 0 | | 0 | | 0 | | 0 | | 1 | | 0 | |
| 1 | Control | 0 | | 0 | | 1 | | 0 | | 1 | | 0 | |
| 1 | Control | 0 | | 0 | | 1 | | 0 | | 1 | | 0 | |
| 1 | Control | 0 | | 0 | | 1 | | 0 | | 1 | | 0 | |
| 1 | Control | 0 | | 0 | | 1 | | 0 | | 1 | | 0 | |
| 1 | Control | 0 | | 0 | | 1 | | 0 | | 1 | | 0 | |
| 1 | CD | 0 | | 0 | | 1 | | 0 | | 1 | | 0 | |
| 1 | UC | 0 | | 0 | | 1 | | 0 | | 1 | | 1 | |
| 1 | CD | 1 | | 0 | | 0 | | 0 | | 1 | | 1 | |
| 1 | CD | 0 | | 0 | | 0 | | 0 | | 1 | | 1 | |
| 1 | aGVHD | 0 | | 1 | | 1 | | 0 | | 1 | | 1 | |
| 1 | CD | 0 | | 1 | | 0 | | 0 | | 1 | | 1 | |
| 1 | CD | 0 | | 1 | | 0 | | 0 | | 1 | | 0 | |
| 2 | UC | 0 | | 0 | | 0 | | 1 | | 1 | | 0 | |
| 2 | UC | 0 | | 0 | | 0 | | 1 | | 1 | | 0 | |
| 2 | UC | 0 | | 0 | | 0 | | 1 | | 1 | | 0 | |
| 2 | aGVHD | 0 | | 0 | | 0 | | 1 | | 1 | | 0 | |
| 2 | ICI/CTLA-4 | 0 | | 0 | | 0 | | 1 | | 1 | | 0 | |
| 2 | aGVHD | 0 | | 0 | | 1 | | 1 | | 1 | | 0 | |
| 2 | CD | 0 | 0 | | 1 | | 1 | | 1 | | 0 | |  |
| 2 | ICI/PD-1^C,P^ | 0 | 0 | | 1 | | 1 | | 1 | | 0 | |  |
| 2 | ICI/PD-1^P^ | 1 | 0 | | 1 | | 1 | | 1 | | 0 | |  |
| 2 | ICI/PD-1 | 0 | 1 | | 0 | | 1 | | 1 | | 1 | |  |
| 2 | UC | 0 | 0 | | 0 | | 1 | | 1 | | 1 | |  |
| 2 | ICI/CTLA-4 | 1 | 1 | | 1 | | 1 | | 1 | | 1 | |  |
| 2 | UC | 0 | 1 | | 1 | | 1 | | 1 | | 1 | |  |
| 2 | ICI/CTLA-4^C,P^ | 0 | 1 | | 1 | | 1 | | 1 | | 0 | |  |
| 2 | ICI/PD-1^C,R^ | 0 | 1 | | 1 | | 1 | | 1 | | 0 | |  |
| 2 | UC | 0 | 1 | | 0 | | 1 | | 1 | | 0 | |  |
| 2 | ICI/CTLA-4 | 1 | 1 | | 0 | | 1 | | 1 | | 0 | |  |
| 3 | ICI/CTLA-4^C,R^ | 0 | 1 | | 0 | | 1 | | 0 | | 1 | |  |
| 3 | UC | 0 | 1 | | 0 | | 1 | | 0 | | 0 | |  |
| 3 | aGVHD | 0 | 1 | | 0 | | 0 | | 0 | | 0 | |  |
| 3 | ICI/PD-1^R^ | 0 | 0 | | 0 | | 1 | | 0 | | 0 | |  |
| 4 | aGVHD | 0 | 0 | | 0 | | 0 | | 0 | | 0 | |  |

CD8+ T, CD4+ T and CD68+ cell infiltration patterns are coded 0 to indicate a scattered/patchy infiltration pattern and 1 to indicate a band-like infiltration pattern in the superficial and deep mucosal layer. Using hierarchical clustering analysis four clusters were identified. ^C^ Corticosteroid resistant. ^R^ Complete response to ICI therapy. ^P^ Partial response to ICI therapy. ICI (immune checkpoint inhibitor), aGVHD (acute graft versus host disease), UC (ulcerative colitis), CD (Crohn’s disease), cytotoxic T-lymphocyte associated protein 4 (CTLA-4), programmed cell death protein 1 (PD-1).
